# Supplementary material for: A tissue-targeted auxin-inducible degradation toolkit for C. elegans embryogenesis
Source: bioRxiv. 2026 Oct 9:2026.10.07.757239. Preprint. [Version 1] doi: 10.64898/2026.10.07.757239 (PMC13692767; doi:10.64898/2026.10.07.757239)
Supplement: Supplementary file 1 [file NIHPP2026.10.07.757239v1-supplement-7.pdf]

bioRxiv preprint doi: https://doi.org/10.64898/2026.10.07.757239; this version posted October 9, 2026. The copyright holder for this preprint (which was not certified by peer review) is the author/funder, who has granted bioRxiv a license to display the preprint in perpetuity. It is made available under a CC-BY 4.0 International license.

SUPPLEMENTARY FIGURES AND FIGURE LEGENDS

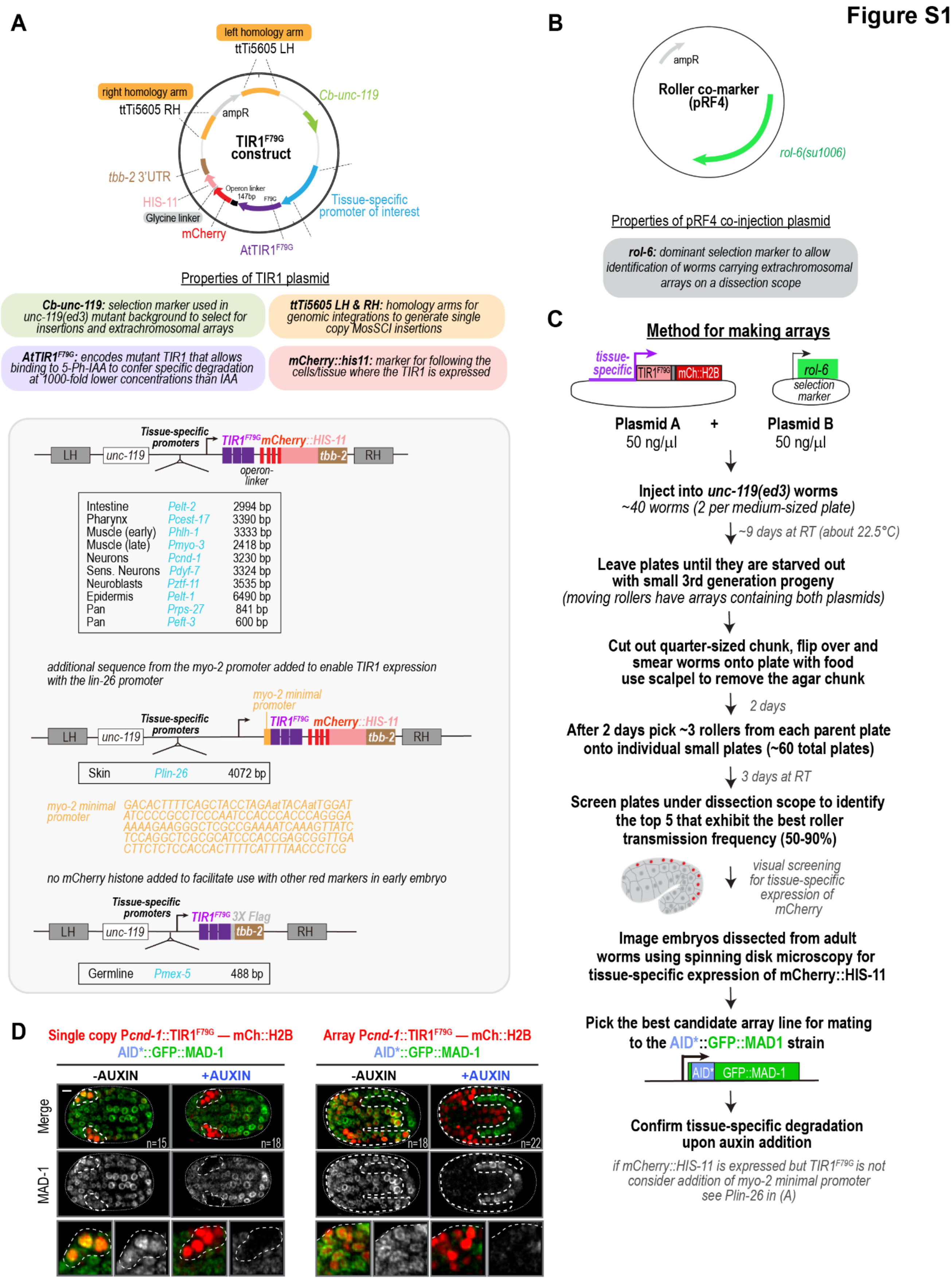

bioRxiv preprint doi: https://doi.org/10.64898/2026.10.07.757239; this version posted October 9, 2026. The copyright holder for this preprint (which was not certified by peer review) is the author/funder, who has granted bioRxiv a license to display the preprint in perpetuity. It is made available under a CC-BY 4.0 International license.

Figure S1. Plasmid design, array construction, and comparison of single-copy versus array expression. (A) Design of the TIR1^{F79G}—mCh::H2B operon expression construct. (*top*) Circular map of the TIR1^{F79G}—mCh::H2B operon plasmid, showing the *Cb-unc-119* selection marker (used in the *unc-119(ed3)* background to select insertions and arrays), the ttTi5605 left and right homology arms (LH, RH) for single-copy MosSCI integration, the tissue-specific promoter of interest, AtTIR1^{F79G} (the mutant TIR1 that binds 5-Ph-IAA derivatives and confers degradation at ~1000-fold lower concentrations compared to IAA), and the mCherry::HIS-11 (mCh::H2B) reporter that marks nuclei in the tissue of where the TIR1^{F79G} is expressed; element properties are summarized below the map. (*middle*) The linear operon structure with sequences corresponding to the tissue-specific promoter— TIR1^{F79G}—the operon linker— mCherry::HIS-11—tbb-2 3' UTR is shown with a table showing the gene names and sizes of the promoter fragments used for expression in the indicated tissues. For the *lin-26* (epidermis) promoter, additional sequence from the *myo-2* minimal promoter as shown was added to enable TIR1^{F79G} expression. **(B)** Map of the pRF4 co-injection plasmid, which carries an ampicillin-resistance marker (ampR) and the dominant *rol-6(su1006)* marker used to identify worms carrying extrachromosomal arrays on a dissecting scope. **(C)** Protocol for generating extrachromosomal-array strains. The TIR1^{F79G}—mCh::H2B operon plasmid (Plasmid A) and the pRF4/*rol-6* selection plasmid (Plasmid B) are co-injected (50 ng/µl each) into *unc-119(ed3)* worms (40 total, two per plate). Plates are grown ~9 days until they starve out with third-generation progeny; a large quarter-sized chunk is cut out from each plate, and the worms are smeared onto food on a new plate. After 2 days, ~3 rollers per parent plate (~60 total) are singled onto individual tiny plates. After 3 days, embryos from the lines with the highest roller transmission (50-90%) are screened by spinning-disk microscopy for tissue-specific mCh::H2B expression, and the best line is mated to the AID*::GFP::MAD1 strain to confirm tissue-specific degradation upon auxin addition. If mCh::H2B is expressed but TIR1^{F79G} is not, adding the *myo-2* minimal promoter fragment upstream of the TIR1 coding region may enable TIR1^{F79G} expression (see the P*lin-26* construct in A). (**D**) Comparison of AID*::GFP::MAD-1 degradation driven by a single-copy insertion of the *Pcnd-1*::TIR1^{F79G}::mCh::H2B operon (*left*) and a *Pcnd-1*::TIR1^{F79G}::mCh::H2B operon containing array (*right*). Embryos from each strain are shown in the absence (−AUXIN, *left*) and presence (+AUXIN, *right*) of 50 µM auxin (5-Ph-IAA-AM). The top row shows merged images with AID*::GFP::MAD-1 in green and mCh::H2B in red. Dashed lines outline the mCh::H2B expressing cells. The middle row shows AID*::GFP::MAD-1 alone in grayscale and zoomed panels on the bottom show a higher-magnification view (~2.5X) of part of the highlighted region from the images above. The single-copy insertion drives neuron-specific degradation, whereas the array drives broad, non-specific degradation across the embryo, which is why the single-copy *Pcnd-1* insertion is recommended for degradation experiments and the array is not (see Fig. 2B). *n* values in the lower right corner of the merged panels are the number of embryos imaged. Scale bar, 5 µm.

bioRxiv preprint doi: https://doi.org/10.64898/2026.10.07.757239; this version posted October 9, 2026. The copyright holder for this preprint (which was not certified by peer review) is the author/funder, who has granted bioRxiv a license to display the preprint in perpetuity. It is made available under a CC-BY 4.0 International license.

Figure S2

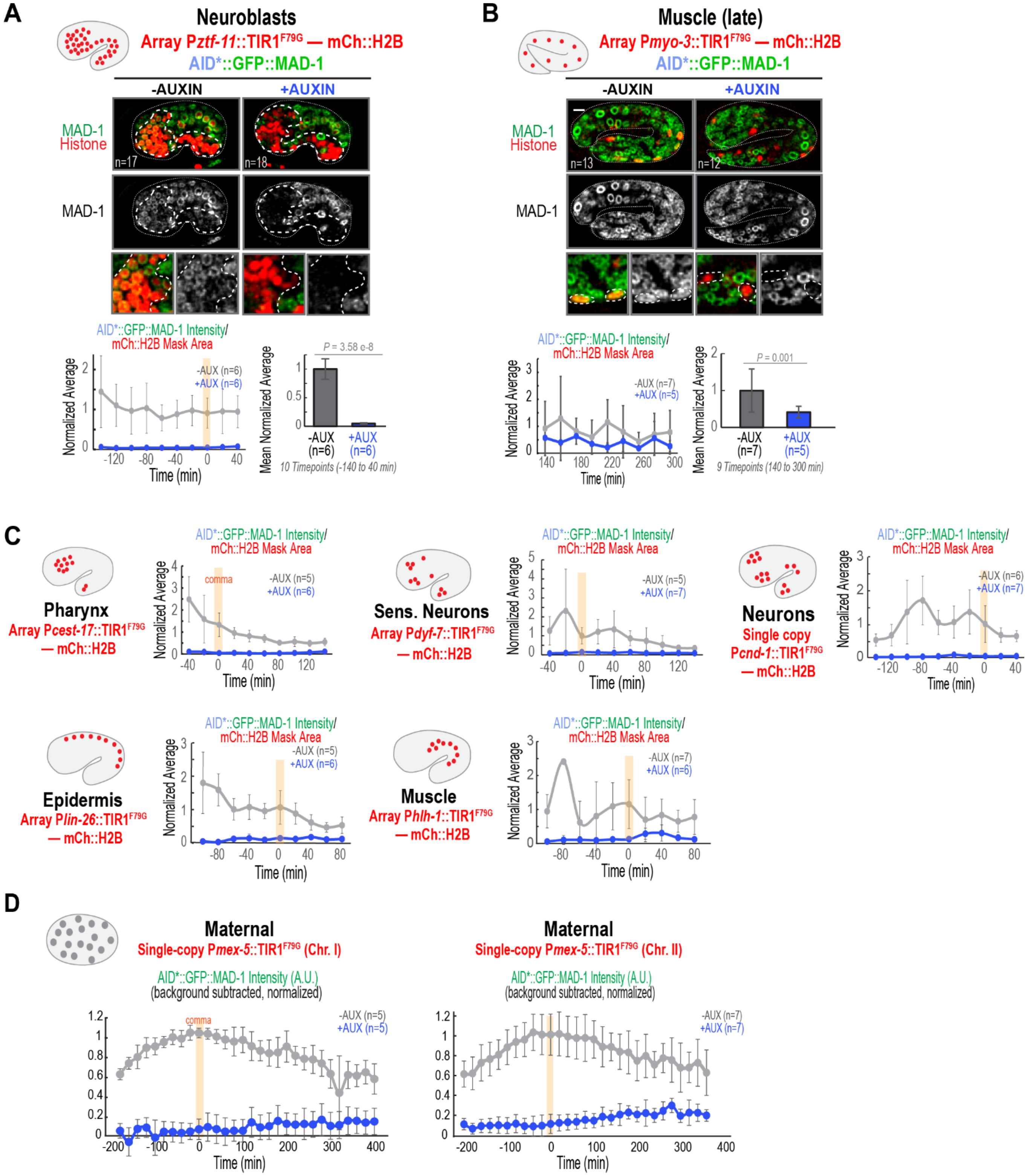

bioRxiv preprint doi: https://doi.org/10.64898/2026.10.07.757239; this version posted October 9, 2026. The copyright holder for this preprint (which was not certified by peer review) is the author/funder, who has granted bioRxiv a license to display the preprint in perpetuity. It is made available under a CC-BY 4.0 International license.

Figure S2. Additional characterization of the tissue-specific auxin-inducible degradation toolkit strains. (A,B) Auxin-mediated degradation of AID*::GFP::MAD-1 in neuroblasts using the P*ztf-11-driven operon* array (A) or in late muscle using the P*myo-3*-driven operon array (B), shown in the absence (*left*) or presence (*right*) of 50 µM auxin (5-Ph-IAA-AM). For each, an embryo schematic indicates the positions of the operon expressing cells. (*top row*) Images show the merge with AID*::GFP::MAD-1 in green and mCh::H2B in red. (*middle row*) Images show AID*::GFP::MAD-1 in grayscale. White dashed lines outline the operon-expressing tissue, defined by the nuclear mCh::H2B reporter. (*bottom row*) Zoomed panels show a higher-magnification view (~2.5X) of regions from the images above. (*bottom, left*) Graph plots normalized AID*::GFP::MAD-1 intensity (AID*::GFP::MAD-1 intensity / mCh::H2B mask area) versus time showing the mean ± SD at each timepoint relative to the comma stage (timepoint 0; gray, −auxin; blue, +auxin). (*bottom, right*) Bar graph (*right*) showing the mean ± SD of the per-embryo averages across the indicated 9 or 10-timepoint windows; quantified as described for Fig. 1D′, E′). *P* values are two-tailed Welch's t-tests comparing the + and − auxin conditions. **(C)** Graphs plot normalized AID*::GFP::MAD-1 intensity (AID*::GFP::MAD-1 intensity / mCh::H2B mask area) versus time showing the mean ± SD at each timepoint relative to the comma stage (timepoint 0; gray, −auxin; blue, +auxin) for operon-expressing embryos from the indicated strains. Data was quantified as in *Fig. 1D',E'* and corresponds to the summary graphs shown in *Fig. 2A.* **(D)** Quantification of AID*::GFP::MAD-1 degradation for the two maternally expressed single-copy P*mex-5* insertions (Chr. I, *left*; Chr. II, *right*), in the absence or presence of 50 µM auxin (5-Ph-IAA-AM). These strains lack the mCh::H2B operon reporter; an embryo schematic summarizes the location of the expressing cells. Plots show the mean ± SD of the background-subtracted, normalized total AID*::GFP::MAD-1 intensity at each timepoint relative to the comma stage (comma = timepoint 0; gray, −auxin; blue, +auxin); see also *Fig. 2A*. *n* is the number of embryos analyzed. Scale bars, 5 µm.

bioRxiv preprint doi: https://doi.org/10.64898/2026.10.07.757239; this version posted October 9, 2026. The copyright holder for this preprint (which was not certified by peer review) is the author/funder, who has granted bioRxiv a license to display the preprint in perpetuity. It is made available under a CC-BY 4.0 International license.

Figure S3

A

Tissue	Promoter	Integration or Array	Chr. #	Recommend?	Parental Strain	MAD-1 Test Strain
Pan tissue	*Peft-3*	Integ.	II	Yes	PLG272	OD6407
Pan-soma (no germline)	*Peft-3*	Integ.	I	Yes	PLG297	OD6467
Pan tissue	*Prps-27*	Integ.	II	Maybe	PLG128	OD6272

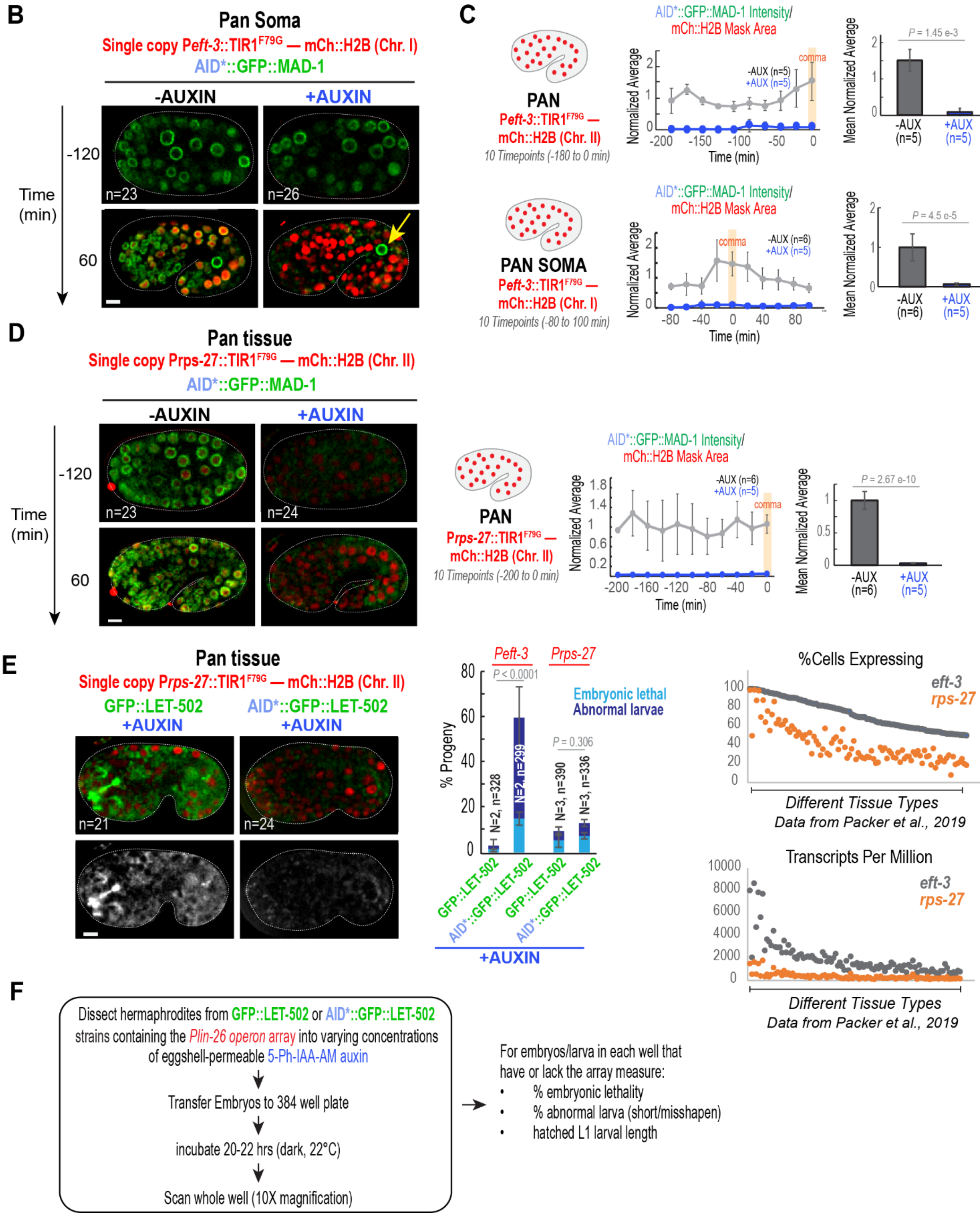

bioRxiv preprint doi: https://doi.org/10.64898/2026.10.07.757239; this version posted October 9, 2026. The copyright holder for this preprint (which was not certified by peer review) is the author/funder, who has granted bioRxiv a license to display the preprint in perpetuity. It is made available under a CC-BY 4.0 International license.

Figure S3. Characterization of pan-tissue degradation strains. (A) Table listing the pan-tissue and pan-soma single-copy integration strains, with target tissue, promoter, integration site (Chr.), whether the strain is recommended, and the parental and AID*::GFP::MAD-1 test strain numbers. **(B)** Timelapse images of embryos expressing AID*::GFP::MAD-1 (*green*) and homozygous for the pan-soma single-copy *Peft-3*::TIR1^{F79G}—mCh::H2B operon insertion (Chr. I; *red*) in the absence (−AUXIN) or presence (+AUXIN) of 50 µM auxin. AID*::GFP::MAD-1 is degraded in the soma but retained in the germline (*yellow arrow*), consistent with germline silencing of this insertion. **(C)** Quantification of AID*::GFP::MAD-1 degradation for the two P*eft-3* insertions: Chr. II ("PAN," *top*) and Chr. I ("PAN SOMA," *bottom*). For each, a schematic (*left*), a plot of normalized AID*::GFP::MAD-1 intensity (AID*::GFP::MAD-1 intensity/ mCh::H2B mask area) versus time (*middle*; mean ± SD at each timepoint relative to the comma stage, timepoint 0; gray, −auxin; blue, +auxin), and a bar graph of the per-embryo averages across the indicated 10-timepoint window (right; mean ± SD) are shown, in the absence or presence of 50 µM auxin. Graphs show different temporal windows because Chr. II PAN expression initiates earlier than the Chr. I PAN, due to differences in germline expression. Quantified as in Fig. 1D′,E′. *P* values, two-tailed Welch's t-test. **(D)** (*left*) Timelapse images of embryos expressing AID*::GFP::MAD-1 (*green*) and homozygous for the pan-tissue single-copy P*rps-27*::TIR1^{F79G}—mCh::H2B operon insertion (Chr. II; *red*) in the absence or presence of 50 µM auxin; AID*::GFP::MAD-1 is degraded throughout. (*right*) Quantification for this strain, presented as in (C) (10-timepoint window, −200 to 0 min; *P* value, two-tailed Welch's t-test). **(E)** (*left*) Images of embryos expressing GFP::LET-502 or AID*::GFP::LET-502 (*green*) and homozygous for the pan-tissue single-copy P*rps-27*::TIR1^{F79G}—mCh::H2B operon insertion (Chr. II; *red*) in the presence of 50 µM auxin; merge (*top*) and GFP alone (*bottom*). AID*::GFP::LET-502 is degraded, including in the sensory depression region. (*middle*) Mean percent of progeny scored as embryonic-lethal (*sky blue*) or abnormal larvae (*navy*) for embryos expressing GFP::LET-502 versus AID*::GFP::LET-502 and homozygous for the pan-tissue P*eft-3* or P*rps-27* operon insertions, in the presence of 50 µM auxin; a significant increase was seen between AID*::GFP::LET-502 and GFP::LET-502 for degradation in the presence of the P*eft-3* but not the P*rps-27* operon. Note that the bars for the P*eft-3* operon insertion are reproduced from Figure 3H for comparison. Error bars, SD; *P* values, chi-square test. (*right*) Single-cell expression data for *eft-3* (*gray*) and *rps-27* (*orange*) across tissue types: percentage of cells expressing and transcripts per million; *eft-3* is expressed more broadly and at higher levels than *rps-27* (data from Packer et al., 2019). **(F)** Scheme of experiment to assess embryonic lethality, larval abnormality, and hatched L1 length across a range of auxin (5-Ph-IAA-AM) concentrations. *n* is the number of embryos/progeny analyzed; *N* is the number of independent experiments. Scale bars, 5 µm.

bioRxiv preprint doi: https://doi.org/10.64898/2026.10.07.757239; this version posted October 9, 2026. The copyright holder for this preprint (which was not certified by peer review) is the author/funder, who has granted bioRxiv a license to display the preprint in perpetuity. It is made available under a CC-BY 4.0 International license.

SUPPLEMENTARY MOVIE LEGENDS

Movie S1. Example movies and quantification of AID*::GFP::MAD-1 in the presence of the P*elt-2* (intestine) and P*elt-1* (epidermal) driven TIR1^{F79G}—mCh::H2B operons. Timelapse images of embryos expressing AID*::GFP::MAD-1 (green), along with arrays expressing the P*elt-2*::TIR1^{F79G}—mCh::H2B (intestine, top) or P*elt-1*::TIR1^{F79G}—mCh::H2B (epidermis, bottom) operons in the presence or absence of 50μM auxin. Each example shows the masked region generated by the red mCh::H2B signal (right panel) and the region within that mask containing green MAD-1 signal (second from the right) that was used to quantify the green intensity signal at each timepoint.

Movie S2. Gallery of toolkit strains. Timelapse images of embryos expressing AID*::GFP::MAD-1 (green), along with the indicated TIR1^{F79G}—mCh::H2B operon expressing arrays (P*elt-2*, P*cest-17*, P*hlh-1,* P*elt-1*, P*lin-26*, P*dyf-7*) or single-copy insertions (P*cnd-1*, P*rps-27*, P*eft-3*, P*mex-5*) in the presence or absence of 50μM auxin**.** Movie pauses briefly to outline the tissues of interest in white for each strain.

Movie S3. *let-502(RNAi)* and *gfp(RNAi) phenotypes*. Timelapse images of embryos expressing AID*::GFP::LET-502 (green). A control embryo is shown along with a *let-502(RNAi)* and a *gfp(RNAi)* embryo as described in Figure 3E.

Movie S4. Epidermal degradation of LET-502. Movie shows timelapse images of embryos expressing AID*::GFP::LET-502 along with the P*lin-26*-driven TIR1^{F79G}—mCh::H2B operon that expresses in the epidermis, in the absence (top) or presence (bottom) of 300 μM auxin. Embryos are the same as those shown in Figure 5D. Fluorescence is overlaid on brightfield images to enable visualization of the changes in embryo morphology throughout development.
